# Adherence to Levothyroxine Treatment Among Patients With Hypothyroidism in Madinah, Saudi Arabia: A Cross-Sectional Study

**DOI:** 10.7759/cureus.40686

**Published:** 2023-06-20

**Authors:** Rasha M Alofi, Lujain S Alrohaily, Raghad A Jan, Saba L Alsaedi, Fai A Mahrous, Mawadah M Alreefi

**Affiliations:** 1 Family Medicine, Ministry of Health in Saudi Arabia, Madinah, SAU; 2 College of Medicine, Taibah University, Madinah, SAU

**Keywords:** drug adherence, saudi arabia, morisky medication adherence scale-8, hypothyroidism, levothyroxine, medication adherence

## Abstract

Background: Hypothyroidism is one of the most prevalent chronic diseases worldwide. The key factor for a good clinical outcome for hypothyroidism is medication adherence, as the mainstay treatment of hypothyroidism is lifelong hormonal replacement therapy, Levothyroxine (LT4). Poor adherence to LT4 is not only linked to great healthcare costs but also to significant economic burdens.

Objectives: The aim of this study is to assess the medication adherence of patients on LT4 treatment in the Madinah region and its association with socio-demographic characteristics, participants’ experience with hypothyroidism and taking LT4, and Morisky Medication Adherence Scale 8-Item (MMAS-8).

Methodology: A cross-sectional study was conducted on 420 hypothyroidism patients on LT4 for at least three months in the Madinah region using a self-administered electronic form. The variables in the questionnaire included socio-demographic characteristics, participants’ experience with hypothyroidism and taking LT4, and MMAS-8.

Results: This study included a total of 420 patients with 81% being females, 52.1% aged 40 years and above, and 91% living in Madinah City. The study shows an overall poor adherence rate toward taking LT4, where the vast majority, 66.7% of the participants, had a low adherence level toward taking LT4, 23.3% had a moderate adherence level, and only 10% had a high adherence level. Results of the multivariate logistic regression showed that the following factors predicted a higher rate of a high level of adherence toward taking levothyroxine, being 50-59 years old, being 60 years or older, and following up regularly in the clinic.

Conclusion: Patients with hypothyroidism showed low adherence to LT4.

## Introduction

Hypothyroidism is one of the most common chronic endocrinological disorders [1]. In the Kingdom of Saudi Arabia (KSA), it accounts for 29.1% of patients who visit primary healthcare centers [2]. Patients who are diagnosed with hypothyroidism need lifelong hormone replacement therapy LT4 [3]. It aims to improve the patient’s condition by maintaining the level of thyroid stimulating hormone (TSH) within the normal range [4].

Adherence to the medication is one of the most important factors to improve the patient’s symptoms. According to World Health Organization (WHO), adherence is defined as “the degree to which the person’s behavior corresponds with the agreed recommendations from a health care provider’’ [5]. Many factors may affect adherence to medications, one of them being the patient’s age. As elderly patients usually suffer from many chronic illnesses that require them to take many medications [2]. Other factors that may affect medication adherence are support from family, good communication between the patient and the doctor, and the financial situation [6].

Until now, only one study in Saudi Arabia measured the perception and medication adherence among patients with primary hypothyroidism [2]. Outside Saudi Arabia, there are many studies that measured LT4 adherence among patients with primary hypothyroidism. A study conducted in Karachi showed that patients with hypothyroidism had moderate adherence to their treatment [3]. Another study that was conducted in Nepal showed more than half of the patients have adhered to the LT4 therapy [7]. One of the most important factors that contribute to good medication adherence is understanding the information on the progression of the disease and its complications [3].

Adherence to hypothyroidism treatment has an effect on the patient’s condition including disease course and outcome [3]. To the best of our knowledge, no previous study has been conducted to assess medication adherence among patients with hypothyroidism in the Madinah region. Therefore, this cross-sectional study aims to assess the medication adherence of patients on LT4 treatment in the Madinah region and its association with socio-demographic characteristics, participants’ experience with hypothyroidism and taking LT4, and MMAS-8.

## Materials and methods

This was an observational cross-sectional study that was conducted in Madinah, Saudi Arabia. It was performed during December 2022-March 2023. The study included 420 participants who were diagnosed with hypothyroidism and taking LT4 treatment for at least three months, both genders, were over 18 years, live in the Madinah region, and could read and understand Arabic or English. A sample size of 384 was estimated using the Qualtrics calculator.

Study measurements

The questionnaire was distributed in Arabic and English using a self-administered electronic form among patients with hypothyroidism in the outpatient clinics of Shuran Primary Healthcare Center. It was also sent specifically to patients with hypothyroidism in the Madinah region through social media. The variables in the questionnaire included socio-demographic characteristics, participants’ experience with hypothyroidism, and taking LT4, and MMAS-8. A license agreement was obtained to use MMAS-8 from © 2006 Donald E. Morisky. The questionnaire contained five different sections. The first part included informed consent to participate in the study. The second part requires answering if they have hypothyroidism or not. The third part requires filling out personal information. The fourth part requires filling out the participants’ experience with hypothyroidism and taking LT4. The last part requires filling out MMAS-8. Participation of hypothyroid patients in the study was based on an informed consent option chosen before the completion of the questionnaire. All participants were assured that all data collected would be confidential and not be used for any purposes except the study. This study was ethically approved by the Research Ethical Committee of Taibah University in Madinah, Saudi Arabia, in December of 2022, study ID TU-013-22 (Reference Number: IORG0008716 - IRB00010413).

Validity and reliability of the questionnaire

MMAS-8 was translated by Apex translation to Arabic, in accordance with ISO 17100-2015. While socio-demographic characteristics and participants’ experience with hypothyroidism and taking LT4 was translated from English to Arabic and back-translated from Arabic to English by two bilingual experts. The translated version from MMAS-8 has been provided to us from © 2006 Donald E. Morisky along with the license to use it. MMAS-8 is a validated and reliable questionnaire that has been used to evaluate patients’ adherence to prescribed medications in many studies [8-10]. The validity and reliability of the Arabic version of MMAS-8 have been tested in a study about medication adherence among patients with type 2 diabetes [11]. MMAS-8 has seven items that are answered with yes or no and one item that has a five-point Likert scale that measures specific medication-taking behavior such as “forgetfulness,” “feeling hassled about sticking to the treatment plan,” or “stopping the regimen because the medication makes the patient feel worse.” The score of MMAS-8 ranged from zero to eight. It classifies adherence into three categories, low adherence (<six), medium adherence (six to seven), and high adherence (=eight) [8].

Data analysis

Data analysis was performed using Statistical Package for the Social Sciences, SPSS 23rd version (IBM Corp., Armonk, NY). Frequency and percentages were used to display categorical variables. Minimum, maximum, mean, and standard deviation were all used to present numerical variables. Independent t-test and analysis of variance (ANOVA) test were used to test for factors associated with adherence scores toward LT4. The ANOVA test was followed by Tukey post-hoc test to determine where the exact difference between groups exists. The level of significance was set at 0.05. Multivariate logistic regression was used to determine factors predicting the high level of adherence to taking levothyroxine. The logistic regression model appropriateness was tested using Omnibus test and Hosmer and Lemeshow test and has proven significant predictability. The level of significance was set at 0.05.

## Results

A total of 420 participants were included in the study. Table 1 shows the socio-demographic profile of the participants. Some 114 (27.1%) of the participants were aged between 18 and 29 years, 87 (20.7%) were aged between 30 and 39 years, 98 (23.3%) were aged between 40 and 49 years, 87 (20.7%) were aged between 50 and 59 years, and 34 (8.1%) were 60 years and older. As for gender, 80 (19%) were males, while 340 (81%) were females. As for the place of residency, 382 (91%) were living in Madinah, 23 (5.5%) were living in Yanbu, six (1.4%) were living in Alhenakiyah, seven (1.7%) were living in Bader, and two (0.5%) were living in other cities.

**Table 1:** Socio-demographic profile of the participants (n = 420).

Demographical characteristics	n	%
Age		
18-29 years	114	27.10
30-39 years	87	20.70
40-49 years	98	23.30
50-59 years	87	20.70
60 years and older	34	8.10
Gender		
Male	80	19.00
Female	340	81.00
Place of residency		
Madinah	382	91.00
Yanbu	23	5.50
Alhenakiyah	6	1.40
Bader	7	1.70
Others	2	0.50

**Figure 1:**
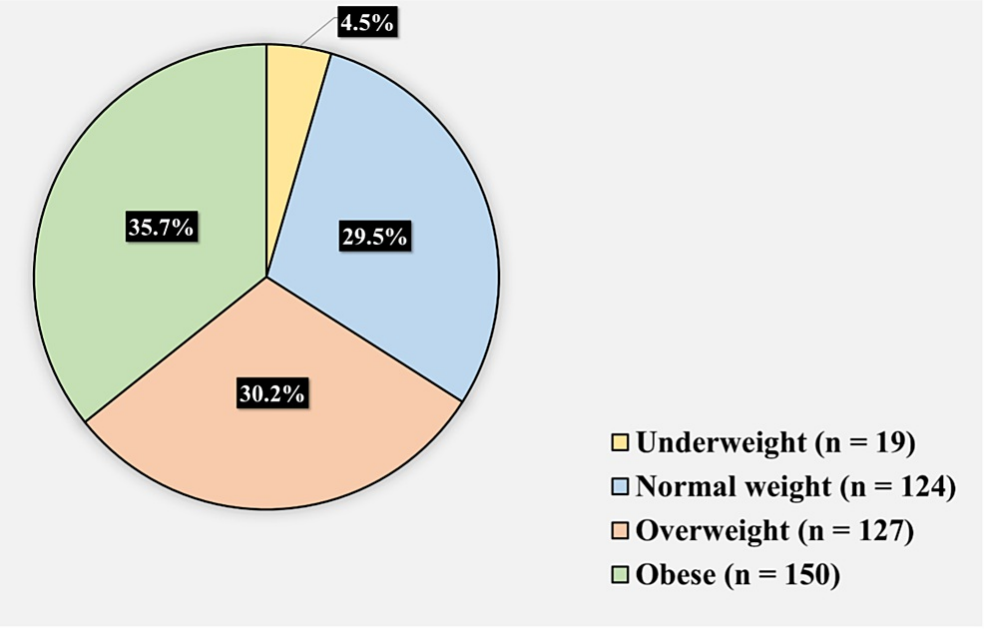
Participants’ BMI. BMI, body mass index

Figure 1 displays the participants’ BMI. 19 (4.5%) of the participants were underweight, 124 (29.5%) of the participants had normal weight, 127 (30.2%) were overweight, and 150 (35.7%) of the participants were obese.

Table 2 shows the participants’ experience with hypothyroidism and taking LT4. 271 (64.5%) of the participants reported regularly following up in the clinic for hypothyroidism, while 149 (35.5%) reported they did not. As for receiving education about hypothyroidism from their managing doctor, 232 (76.9%) reported they received an education, while 97 (23.1%) reported they did not. As for receiving education about LT4 from their managing doctor, 288 (68.6%) reported they received an education, while 132 (31.4%) reported they did not. As for the duration of LT4 prescription, 105 (25%) reported taking LT4 for 3 months-1 year, 119 (28.3%) reported taking LT4 for 1-5 years, and 196 (46.7%) reported taking LT4 for more than 5 years. Some 192 (45.7%) reported obtaining LT4 by buying from pharmacies, while 228 (54.3%) reported obtaining it free from the clinic. As for needing assistance in taking medication, 86 (20.5%) reported they did need assistance, while 334 (79.5%) reported they did not.

**Table 2:** Participants' experience with hypothyroidism and taking levothyroxine.

Question	n	%
Q1/ Do you follow up on your condition regularly in a clinic?		
Yes	271	64.5
No	149	35.5
Q2/ Have you received education about hypothyroidism from your managing doctor?		
Yes	323	76.9
No	97	23.1
Q3/ Have you received education about Levothyroxine from your managing doctor?		
Yes	288	68.6
No	132	31.4
Q4/ Duration of Levothyroxine prescription?		
3 months-1 year	105	25
1-5 years	119	28.3
More than 5 years	196	46.7
Q5/ From where did you obtain your Levothyroxine?		
Bought from pharmacies	192	45.7
Free from the clinic	228	54.3
Q6/ Do you need assistance in taking medication?		
Yes	86	20.5
No	334	79.5

**Figure 2:**
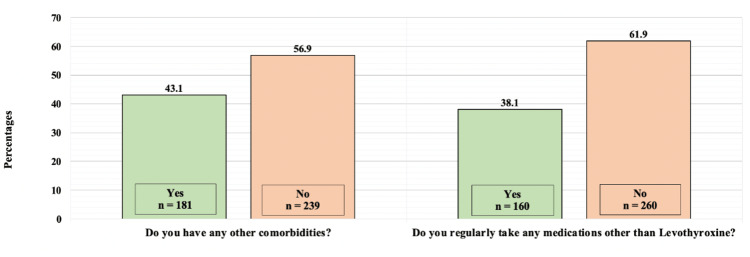
Participants’ history of having other co-morbidities and using medications other than Levothyroxine.

Figure 2 shows participants' history of having other co-morbidities and using medications other than LT4. Some 181 (43.1%) of the participants reported having other comorbidities, while 239 (56.9%) reported they do not. As for regularly taking medication other than LT4, 160 (38.1%) reported they did, while 260 (61.9%) reported they did not. 

Table 3 shows the assessment of participants’ adherence toward taking LT4 using the Morisky Medication Adherence Scale 8-Item (MMAS-8). The minimum adherence score was zero, the maximum was eight, and the mean was 4.61 + 2.1. 

**Table 3:** Assessment of participants’ adherence toward taking Levothyroxine using MMAS-8. © 2006 Donald E. Morisky MMAS-8, Morisky Medication Adherence Scale 8-Item

Question	n	%
Q1/ Do you sometimes forget to take your [health concern] medication(s)?		
Yes	249	59.3
No	171	40.7
Q2/ People sometimes miss taking their medications for reasons other than forgetting. Thinking over the past two weeks, were there any days when you did not take your [health concern] medication(s)?		
Yes	200	47.6
No	220	52.4
Q3/ Have you ever cut back or stopped taking your medication(s) without telling your doctor, because you felt worse when you took it?		
Yes	143	34
No	277	66
Q4/ When you travel or leave home, do you sometimes forget to bring along your [health concern] medication(s)?		
Yes	208	49.5
No	212	50.5
Q5/ Did you take your [health concern] medication(s) yesterday?		
No	93	22.1
Yes	327	77.9
Q6/ When you feel like your [health concern] is under control, do you sometimes stop taking your medication(s)?		
Yes	134	31.9
No	286	68.1
Q7/ Taking medication(s) every day is a real inconvenience for some people. Do you ever feel hassled about sticking to your [health concern] treatment plan?		
Yes	266	63.3
No	154	36.7
Q8/ How often do you have difficulty remembering to take all your medication(s)?		
All the time	19	4.5
Usually	32	7.6
Sometimes	115	27.4
Once in a while	121	28.8
Never-Rarely	133	31.7
Adherence Score (lowest possible = 0, highest possible = 8)		
Minimum	0	
Maximum	8	
Mean	4.61	
Standard deviation	2.1	

**Figure 3:**
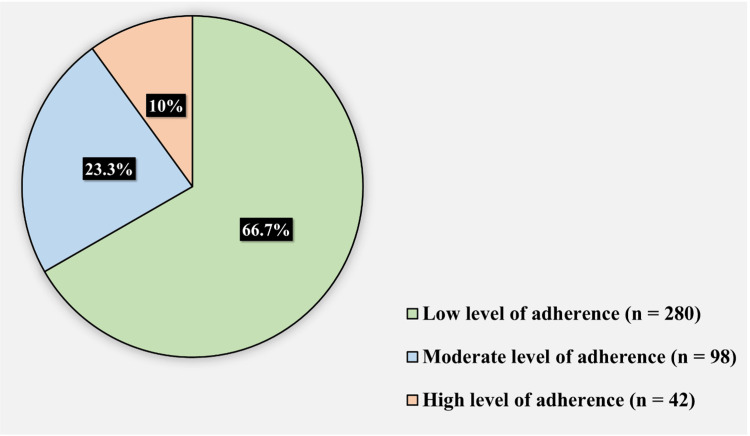
Participants’ adherence levels toward taking Levothyroxine.

Figure 3 shows the participants’ adherence levels toward taking LT4. Some 280 (66.7%) of the participants had a low level of adherence toward taking LT4, 98 (23.3%) had a moderate level of adherence toward taking LT4, and 42 (10%) had a high level of adherence toward taking LT4.

Table 4 shows the factors associated with adherence toward taking LT4. Age was significantly associated with adherence score toward taking LT4 (p < 0.001), where it was observed that the older the age group, the higher the adherence score. Tukey post-hoc test revealed that those aged 18-29 years old had a significantly lower adherence score compared to those from the age group 40-49 years, 50-59 years, and those from the age group 60 years and older respectively. Moreover, Tukey post-hoc test revealed that those aged 30-39 years had a significantly lower adherence score compared to those aged 50-59 years, and those aged 60 years and older respectively. The city was also significantly associated with adherence score toward LT4 (p = 0.015), where it was observed that those from Madinah had a significantly higher adherence score compared to those from Yanbu (4.73 + 2.16 vs 3.6 + 2.22). Following up regularly in a clinic was also significantly associated with adherence score toward taking LT4 (p < 0.001), where it was observed that those following up regularly in a clinic had a significantly higher adherence score compared to those who did not (4.89 + 2.19 vs 4.1 + 2.11). Duration of taking LT4 was also significantly associated with adherence score toward taking LT4 (p < 0.001), where it was observed the higher the duration of taking LT4, the higher the adherence score of taking LT4. Tukey post-hoc test revealed that those who reported taking LT4 for more than five years had a significantly higher adherence score compared to those who reported taking LT4 for 3 months-1 year and compared to those taking LT4 for 1-5 years respectively. Mean from obtaining LT4 was also significantly associated with adherence score toward taking LT4 (p = 0.003), where it was observed that those who bought the medication from pharmacies had a significantly higher adherence score compared to those who took it free from the clinic (4.95 + 2.12 vs 4.32 + 2.21). Needing assistance in taking medication was also significantly associated with adherence score toward taking LT4 (p = 0.019), where it was observed that those needing assistance had a significantly lower adherence score toward taking LT4 compared to those who did not need assistance (4.12 + 2.23 vs 4.74 + 2.16). Gender, body mass index (BMI), receiving education about hypothyroidism, receiving education about LT4, having other comorbidities, and regularly taking other medications, were all not significantly associated with adherence scores toward taking LT4. 

**Table 4:** Factors associated with adherence toward taking Levothyroxine. *Significant at level 0.05

Factor	Adherence score		p-Value
	Mean	Standard deviation	
Age			< 0.001*
18-29 years	3.87	2.04	
30-39 years	4.15	2.19	
40-49 years	4.71	1.97	
50-59 years	5.49	2.13	
60 years and older	5.73	2.25	
Gender			0.378
Male	4.42	2.32	
Female	4.66	2.16	
City			0.015*
Madinah	4.73	2.16	
Yanbu	3.60	2.22	
BMI			0.128
Underweight	3.93	2.10	
Normal weight	4.72	2.11	
Overweight	4.34	2.34	
Obese	4.84	2.11	
Do you follow up on your condition regularly in a clinic?			< 0.001*
Yes	4.89	2.19	
No	4.10	2.11	
Have you received education about hypothyroidism from your managing doctor?			0.340
Yes	4.55	2.17	
No	4.80	2.25	
Have you received education about Levothyroxine from your managing doctor?			0.828
Yes	4.63	2.16	
No	4.58	2.26	
Duration of Levothyroxine prescription?			< 0.001*
3 months-1 year	3.75	2.09	
1-5 years	4.37	2.16	
More than 5 years	5.22	2.08	
From where you obtain your Levothyroxine?			0.003*
Bought from pharmacies	4.95	2.12	
Free from the clinic	4.32	2.21	
Do you need assistance in taking medication?			0.019*
Yes	4.12	2.23	
No	4.74	2.16	
Do you have any other comorbidities?			0.663
Yes	4.56	2.24	
No	4.65	2.16	
Do you regularly take any medications other than Levothyroxine?			0.104
Yes	4.83	2.21	
No	4.47	2.17	

Table 5 shows the multivariate logistic regression (factors predicting the high level of adherence to taking Levothyroxine). The logistic regression model included the following variables: age, gender, city, BMI, regular follow-up in the clinic, receiving education about hypothyroidism, receiving education about levothyroxine, duration of levothyroxine prescription, place of obtaining levothyroxine, needing assistance in taking medication, having other co-morbidities, and taking other medications regularly. The following factors predicted a higher rate of the high level of adherence toward taking levothyroxine, being 50-59 years old (p < 0.001, odds ratio = 20.59), being 60 years or older (p = 0.001, odds ratio = 26.36), and following up regularly in the clinic (p = 0.001, odds ratio = 5.99).

**Table 5:** Multivariate logistic regression (factors predicting the high level of adherence to taking Levothyroxine). *Significant at level 0.05

Factor		p-Value	Odds ratio	Confidence interval	
Age (18-29 years is the referent)					
30-39 years		0.127	4.06	0.67	24.54
40-49 years		0.144	3.73	0.64	21.84
50-59 years		< 0.001*	20.59	3.82	110.87
60 years and older		0.001*	26.36	4.16	167.07
Gender (Male vs Female)		0.214	1.84	0.71	4.79
City (Medinah vs Yanbu)		0.685	1.56	0.18	13.30
BMI (underweight is the referent)					
Normal weight		0.191	0.19	0.02	2.30
Overweight		0.644	0.57	0.05	6.26
Obese		0.321	0.29	0.03	3.33
Do you follow up on your condition regularly in a clinic? (yes vs no)		0.001*	5.99	2.05	17.53
Have you received an education about hypothyroidism from your managing doctor? (yes vs no)		0.207	0.49	0.16	1.48
Have you received education about Levothyroxine from your managing doctor? (yes vs no)		0.355	1.64	0.57	4.70
Duration of Levothyroxine prescription (3 months-1 year is the referent)					
1-5 years		0.324	1.82	0.56	5.94
More than 5 years		0.267	1.85	0.62	5.47
From where do you obtain your Levothyroxine? (bought from pharmacies vs free from the clinic)		0.462	1.34	0.62	2.91
Do you need assistance in taking medication? (yes vs no)		0.180	0.46	0.15	1.43
Do you have any other comorbidities? (yes vs no)		0.069	0.39	0.14	1.08
Do you regularly take any medications other than Levothyroxine? (yes vs no)		0.283	1.71	0.64	4.57

## Discussion

The current study shows an overall poor adherence rate toward taking LT4, where the vast majority, 66.7% of the participants, had a low adherence level toward taking LT4. Age, city of residency, following up regularly, duration of taking LT4, mean for obtaining LT4, and needing assistance in taking LT4 were all significantly associated with adherence scores toward taking the medication. Results of the multivariate logistic regression showed that the following factors predicted a higher rate of a high level of adherence toward taking levothyroxine, being 50-59 years old (p < 0.001, odds ratio = 20.59), being 60 years or older (p = 0.001, odds ratio = 26.36), and following up regularly in the clinic (p = 0.001, odds ratio = 5.99).

In this study, the vast majority, 66.7% of the participants, had a low adherence level toward taking LT4, 23.3% had a moderate adherence level, and only 10% had a high adherence level. These results come in agreement with the statistics of El Helou et al. in their study that was conducted in Lebanon where it showed that 54.9% of the participants had low adherence levels toward taking LT4, and a rate of 30.6% and 14.5% for the patients having medium adherence levels and high adherence levels toward taking LT4 respectively [12]. In contrast to these results, another study by Cappelli et al. conducted in Italy has revealed that only 1.9% of the studied population were classified as low adherers toward LT4, 10.9% as medium adherers, and 87.2% as high adherers, a significantly higher rate [4]. Moreover, Crilly and Esmail conducted a study in England, which reports 78% adherence levels toward medications among their participants [13]. This discrepancy of findings across different geographical regions may suggest that medication non-adherence is an issue that may be more prevalent among Middle East countries and that demographic and cultural factors may affect adherence which requires further in-depth study.

In this study, age was significantly associated with adherence toward taking LT4, where it was observed that the older the age group, the higher the adherence score, and that those aged 18-29 years old had significantly lower adherence scores compared to other age groups. As for gender, it was shown that gender exerted little influence on adherence levels among this study’s population. These findings come in consistence with the longitudinal study of Briesacher et al. that compared drug adherence rates among patients with seven different medical conditions including hypothyroidism, where it revealed improving adherence rates with increasing age, particularly in patients with hypertension, type 2 diabetes, and hypothyroidism [14]. Moreover, their statistics showed that in the comparison of adherence by gender, adherence rates showed little variation [14]. However, studies by Alluhayyan et al. and El Helou et al. have observed that older patients and males were found to be less adherent to LT4 [2, 12]. This variation in the findings provided can be justified by the fact that elderly subjects tend to have comorbidities which can either have a positive impact on medication adherence by getting used to taking medications or can have a negative impact by affecting cognitive skills and memory.

In this study, participants who followed up regularly in a clinic had a significantly higher adherence score compared to those who did not. This finding is consistent with the studies of Alluhayyan et al. and El Helou et al. where a statistically significant inverse association was found between patients’ adherence to postponing physician visits [2, 12]. As in these studies, it was observed that patients who postpone their follow-ups had lower adherence [2, 12]. Moreover, Shakya Shreshta et al. have reported similar findings as missed follow-ups were significantly associated with medication non-compliance [7]. This can be understood as patients who are reluctant toward their medical appointments for follow-ups, are more prone to be reluctant to take their medications as well as to follow instructions from their physicians concerning their clinical status, medication dosage, and actions for managing their conditions [15].

Studies analyzing the adherence level toward LT4 medication and its associative factors among residents of Saudi Arabia are scarce. Thus, this study enriches the literature with the status of the adherence level among hypothyroidism patients in the Madinah Region, which is one of the largest regions in Saudi Arabia. Furthermore, the MMAS-8 questionnaire used to evaluate patient adherence to LT4 is a validated and reliable questionnaire that has been used to evaluate patients’ adherence to prescribed medications in many studies [8-10]. However, the study’s targeted population was residents of the Madinah region including Madinah, Yanbu, Alhenakiya, Bader, and other cities. More than 90% of the participants were living in Madinah, hence, the extent of generalization for this study’s results is restricted.

## Conclusions

Our study demonstrated low adherence to LT4. Improving patients' adherence to medication will improve their quality of life and decrease the economic burden that results from non-compliance with medications in patients with chronic medical conditions like hypothyroidism. To overcome this low adherence score, it is recommended to implement educational programs for hypothyroidism patients to increase the awareness level about the importance of medication adherence in the clinical outcome and disease course. Furthermore, the doctor-patient relationship should be enhanced to encourage patients to participate in their own medical care, increasing the likelihood of medication compliance.
